# Certificates as to Extra-Professional Cures

**Published:** 1916-01

**Authors:** 


### Certificates as to Extra-Professional Cures.

The popular press is replete with statements, some under oath, as to the reliability of various nostrums. The **Journal of the A. M. A.** has done excellent service in showing up some of these testimonials, for instance in publishing the death certificates of patients advertised as cured of consumption. Some time ago, one of our daily papers published a testimonial from a prominent Buffalo physician whose name was not found in either telephone book nor in the directory and who was not known to any of several members of the profession interrogated. The attention of the editor of the paper publishing the advertisement was called to the matter and it was finally explained that the doctor in question had, some years previously, spent a few months in Buffalo, as an employee of the advertisers.

We suggest that, in each locality, the profession systematically investigate every advertisement of claims of cure by nostrums or any definite attack on the profession itself. By co-operation, throughout the country, definite statements could be met with definite statements and the unfavorable influence of advertising false claims, would be materially checked.

The BUFFALO MEDICAL JOURNAL will co-operate with any such professional movement and will endeavor to secure the general co-operation of the medical press.

